# A Novel Method to Quantify Near-Surface Boundary-Layer Dynamics at Ultra-High Spatio-Temporal Resolution

**DOI:** 10.1007/s10546-022-00752-3

**Published:** 2022-11-19

**Authors:** Michael Haugeneder, Michael Lehning, Dylan Reynolds, Tobias Jonas, Rebecca Mott

**Affiliations:** 1 WSL-Institute for Snow and Avalanche Research SLF, Davos, Switzerland; 2 School of Architecture, Civil and Environmental Engineering, École Polytechnique Fédérale de Lausanne, Lausanne, Switzerland

**Keywords:** Two-dimensional atmospheric measurements, Infrared thermography, Patchy snow cover, Snow melt, Surface–atmosphere interaction

## Abstract

The lateral transport of heat above abrupt (sub-)metre-scale steps in land surface temperature influences the local surface energy balance. We present a novel experimental method to investigate the stratification and dynamics of the near-surface atmospheric layer over a heterogeneous land surface. Using a high-resolution thermal infrared camera pointing at synthetic screens, a $30\,\hbox {Hz}$ sequence of frames is recorded. The screens are deployed upright and horizontally aligned with the prevailing wind direction. The screen’s surface temperature serves as a proxy for the local air temperature. We developed a method to estimate near-surface two-dimensional wind fields at centimetre resolution from tracking the air temperature pattern on the screens. Wind field estimations are validated with near-surface three-dimensional short-path ultrasonic data. To demonstrate the capabilities of the screen method, we present results from a comprehensive field campaign at an alpine research site during patchy snow cover conditions. The measurements reveal an extremely heterogeneous near-surface atmospheric layer. Vertical profiles of horizontal and vertical wind reflect multiple layers of different static stability within $2\,\hbox {m}$ above the surface. A dynamic, thin stable internal boundary layer (SIBL) develops above the leading edge of snow patches protecting the snow surface from warmer air above. During pronounced gusts, the warm air from aloft entrains into the SIBL and reaches down to the snow surface adding energy to the snow pack. Measured vertical turbulent sensible heat fluxes are shown to be consistent with air temperature and wind profiles obtained using the screen method and confirm its capabilities to investigate complex in situ near-surface heat exchange processes.

**Supplementary Information:**

The online version contains supplementary material available at 10.1007/s10546-022-00752-3.

## Introduction

A precise understanding of the seasonal snow cover dynamics is of crucial importance for various applications (Lehning 2013). Snow melt and its timing have impacts on the ecosystem (Wheeler et al. 2016), the climate and water availability in cold regions (Cohen and Rind 1991; Chapin et al. 2005; Beniston et al. 2018), and provides drinking water to downstream communities (Sturm et al. 2017). Hydrological forecasts for flood prevention (Wever et al. 2017) or prediction of the hydropower potential (Schaefli et al. 2007) rely on solid knowledge about the snow cover and its spatio-temporal dynamics.

An important driver for snow cover variations is the interaction of the snow surface and the adjacent atmosphere in the form of mass and energy fluxes (Mott et al. 2018). The energy exchange becomes especially important when the snow cover becomes patchy. Incoming shortwave radiation and a lower surface albedo leads to a warming of bare ground. Melting snow patches, however, remain at $0\,^\circ \hbox {C}$. The resulting strong spatial heterogeneity of surface temperatures interacts with the atmospheric flow at different scales and increases the turbulent exchange of sensible and latent heat towards the snow cover (Liston 1995; Mott et al. 2013; Letcher and Minder 2017; Schlögl et al. 2018b). The crucial question in this context becomes: how much of the laterally advected heat reaches the snow surface and leads to an input in the snow pack’s energy balance (Ménard et al. 2014; Mott et al. 2015; Harder et al. 2017; Mott et al. 2017)? Furthermore, interactions between the atmospheric flow field and heat exchange processes over the melting surface occur not only in spring snowmelt, but also influence the melt of glaciers and, thus, their mass balance (Mott et al. 2020).

Few experimental campaigns have been carried out to assess this research question. Granger et al. (2006) measured vertical profiles of air temperatures over bare ground and snow patches. Using their data, they give a best-fit approximation for the growth of a stable internal boundary layer developing over the snow surface (Garratt 1990). Mott et al. (2016) conducted wind-tunnel experiments to study the influence of the topography on the atmosphere close to the snow surface. They point to local decoupling in weak wind conditions. The effect of topography on the local wind speed and, thus, the energy balance of the melting snow pack is also found to be significant by Fujita et al. (2010). They investigated the melt of a persistent snow patch over four decades and related it to meteorological observations. Later, Mott et al. (2017) found evidence for atmospheric decoupling in a comprehensive field campaign including various turbulence measurements. Furthermore, the authors point to the importance of the wind on the exchange of sensible heat with the snow surface through the development of internal boundary layers. In an effort to quantify the advection of both sensible and latent heat over snow patches, Harder et al. (2017) measured vertical temperature and humidity profiles at specific locations over the transition from bare ground to snow. They conclude that advected sensible heat has a significant contribution to the energy balance of a melting snow patch. Advection of latent heat, however, strongly depends on the bare soil moisture.

With the available experimental data, several approaches to model the advection of heat and the resulting turbulent heat fluxes have been developed. Essery et al. (2006) and Granger et al. (2002) used a boundary-layer integration model to estimate aerial averages of advected heat by integrating over the temperature difference between an upwind (over bare ground) temperature profile and a profile over the snow patch. Sauter and Galos (2016) performed large-eddy simulations (LES) in order to better represent the small-scale turbulent processes close to the snow surface. Later, Schlögl et al. (2018a) developed a temperature footprint approach to estimate the influence of air temperatures close to the surface in the upwind fetch of a grid point. However, modelling the effect of lateral heat advection on the snow melt over a large-scale domain remains an unresolved issue in snow-hydrological modelling.

To address this problem and gain deeper process understanding of snow–atmosphere interactions and feedbacks, further measurements on the sub-metre-scale are necessary. Thermal infrared imaging of a vertical synthetic screen to investigate the near-surface atmosphere can fill this gap. With spatially continuous data across the transition from bare ground to snow, the shortcomings of point-based measurements can be overcome. Grudzielanek and Cermak (2015) used a similar set-up to investigate nocturnal katabatic and drainage flows. For this study, we further improved the set-up and applied it on a patchy snow cover. With that we are able to retrieve high spatio-temporal resolution air temperature measurements within the atmospheric layer adjacent to the surface. On the basis of Inagaki et al. (2013), who used a time series of thermal images of a sunlit house wall to investigate air motion close to the wall, we developed an algorithm to estimate near-surface wind speeds. In combination with the measured air temperatures, this allows assessment of both the static stratification and the extreme boundary-layer dynamics in the first few metres above the land surface.

In the following chapter, the experimental set-up and the data processing of the thermal infrared data are described. Subsequently, to show the capabilities of the method, the near-surface atmosphere over a patchy snow cover is described qualitatively using an infrared frame. We present a new method for retrieving vertical profiles of the horizontal and vertical wind speed showing the extreme dynamics of the near-surface atmospheric layer. Finally, the estimated wind fields are compared to both reference measurements and calculated turbulent vertical sensible heat fluxes.

## Methods

**Fig. 1:**
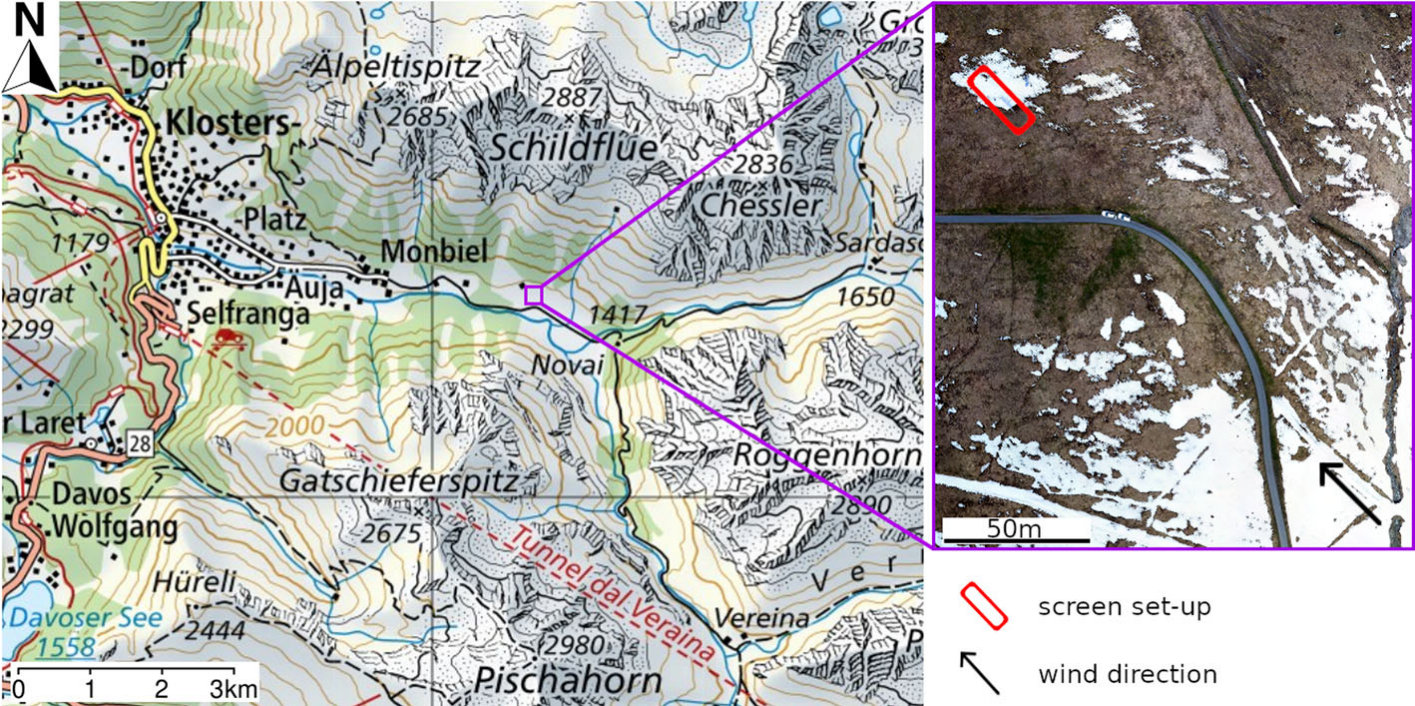
The measurement site in the alpine valley of Monbiel (Klosters, Switzerland). On the right a UAV image shows the position of the screen set-up with the upwind fetch. The snow cover fraction of the area shown in the UAV image is 0.25. Topographic map taken from map.geo.admin.ch

Experimental data for the analysis on near-surface atmospheric layer dynamics were collected during a comprehensive field campaign in late spring 2021 in the alpine valley of Monbiel (Klosters, Switzerland). Figure 1 shows a map of the investigation area along with an image captured by an unmanned aerial vehicle (UAV).

Measurements were taken on the slightly south-exposed valley floor at a height of 1360 $\hbox {m}$ above sea level. The UAV image in Fig. 1 depicts the snow cover at the time of the measurements used for this study. The total snow cover fraction of the area shown in the UAV image is 0.25. However, the snow cover fraction decreases from south-east to north-west due to the slightly inclined, south-exposed slope in the upper part of the image. On the upwind edge of a snow patch with a size of $20\, \times 15\,\hbox {m}^{2}$ and a maximum snow depth of $h_s = 0.3\,\hbox {m}$, the screen set-up was deployed. The snow in the surrounding areas mostly disappeared except for some small patches. The bare ground mainly consisted of alpine meadow with a small paved road 60-m upwind of the screens. The screen method has been developed and tested on multiple different infrared sequences. However, as the primary aim is to demonstrate the capabilities of the screen method, evaluations in this publication stem from a 10-min infrared sequence recorded on 28 April 2021 1200 LT (local time = UTC + 2h). During the recorded period, multiple gusts advected warm air over the snow surface. Furthermore, measurements of the short-path ultrasonic anemometer close to the screen are available for this period for validation (see Sect. 3.3). In future research, we plan to analyse the full dataset to more quantitatively assess processes of near-surface heat exchange.

In the following, the screen set-up utilizing a thermal infrared camera is introduced in Sect. 2.1. Subsequently, Sect. 2.2 describes the data evaluation including a detailed process scheme. Finally, Sect. 2.3 gives a brief insight into the conducted turbulence measurements.

### Screen Set-Up

**Fig. 2:**
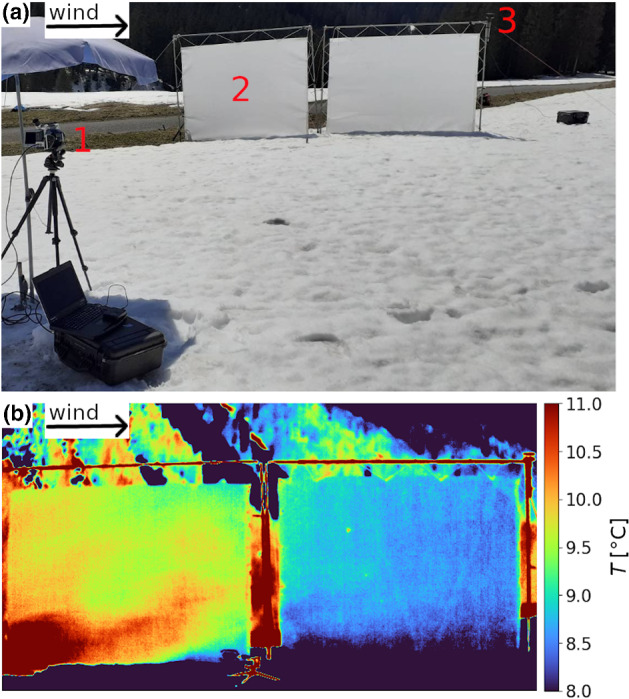
**a** Screen set-up used to obtain highly spatio-temporally resolved thermal infrared pictures. (1) Sun-shielded thermal infrared camera, (2) stretched thin synthetic screens, (3) two-dimensional ultrasonic anemometer. **b** Example for a recorded infrared frame. The screen’s surface temperature serves as a proxy for local air temperature and is shown according to the colour bar on the right

The screen set-up (Fig. 2a) was adapted from Grudzielanek and Cermak (2015), who used it to investigate katabatic flow properties.

In order to record a sequence of high spatio-temporal resolution thermal infrared frames, a sun-shielded thermal infrared camera (InfraTec VarioCam HD) was deployed on a tripod and connected to a laptop for recording (marked with ‘1’ in Fig. 2a). It points at two vertical screens aligned with the wind direction over the transition of bare ground to snow covered areas (‘2’). The screens are each 2.8-m long and 1.9-m high (for further description see Sect. 2.1.2). A parallel alignment of the screen axis with the wind direction is crucial to avoid artificial vertical movement of air impinging upon the screen for non-parallel flows. Figure 2b shows a single infrared frame as an excerpt from a sequence recorded on 23 April 2021 in Monbiel. In the following, the abbreviations ‘TIR camera’ for thermal infrared camera and ‘infrared frame’ for a single frame out of the 10-min sequence are used. As shown in Fig. 2a, bare ground (mainly alpine meadow) covers the surface in the upwind fetch of the investigated snow patch. The UAV image in Fig. 1 gives an overview of the upwind surface. The area around the screens downwind of the transition is snow covered. The screens cover a wind fetch distance over snow of approximately 5 m. The surface temperature of the screen serves as a proxy for air temperature (Grudzielanek and Cermak 2015) and is shown in Fig. 2b according to the colour bar. The example infrared frame shows a warm air plume advected by the wind reaching about 1.5 m over the snow patch, while there is still a thin layer of cold air close to the snow surface. The air further downwind is significantly colder. In the background a partly snow covered, forested ridge is visible.

#### Thermal Infrared Camera

High spatio-temporal resolution thermal frames are recorded by a TIR camera. An uncooled microbolometer in the TIR camera measures the emitted spectral power in a wavelength interval $7.5\,\upmu \hbox {m} \le \lambda \le 14\,\upmu \hbox {m}$. To relate the measured spectral power to the surface temperature $T_s$, the Stefan–Boltzmann law:

1 $$\begin{aligned} \Phi = \epsilon \sigma T_s^4, \end{aligned}$$

is used. The emitted power per area is denoted by $\mathrm {\Phi }$, the emissivity of the emitting object by $\epsilon$, and the Stefan–Boltzmann constant $\sigma \approx 5.67 \times 10^{-8}\,\hbox {W}\, \hbox {m}^{-2}\,\hbox {K}^{-4}$. The emissivity $\epsilon$ is introduced as a material-specific constant. It describes how well the object can be approximated as a perfect black body with an emissivity $\epsilon = 1$. If $\epsilon < 1$, a further correction accounting for reflection and transmission of the surrounding environment can be applied to Eq. 1. The screens used in this study have an emissivity of $\epsilon _{screen}=0.94$ (Grudzielanek and Cermak 2015). Due to strong thermal heterogeneities in the surrounding of the screen, further inaccuracies due to transmission and reflection are likely and cannot be corrected. The influence of the atmosphere between the TIR camera and the screens on the transmitted infrared signal is neglected due to the short distance. Furthermore, the analysis in this study only evaluates relative differences in the screen temperatures. Therefore, no corrections are applied.

The detection of surface temperatures with the TIR camera is subject to different sources of artefact(s). The most relevant for this study is vignetting. Vignetting is caused by a decrease in illumination of the sensor with increasing off-axis distance due to the optical system (Li and Zhu 2009). In the data, the effect is visible as a temperature gradient from the centre towards the periphery of the field of view. Vignetting gets stronger with stronger temperature changes. Its impact is limited by a shutter inside the TIR camera that is moved in front of the microbolometer covering the whole field of view for a fraction of a second. Using the known temperature of the shutter, the microbolometer is recalibrated every 30 s. In the field, it is important that the TIR camera has adapted to ambient temperature before starting to record. To minimize the influence of shortwave radiation on the TIR camera, an umbrella was used as sun protection (see Fig. 2a). Furthermore, the preprocessing steps (see Sect. 2.2.1), and in particular the subtraction of a back looking time average, help to reduce the effects on the recorded temperature field.

For this study, we used an InfraTec VarioCAM HD offering a spatial resolution of $1024 \times 768\,\textrm{pixels}$ to capture boundary-layer dynamics. The TIR camera records infrared frames at 30 Hz. One infrared sequence contains 18,000 frames covering 10 min.

#### Screens and Wind Direction Measurement

The screens consist of a stretched, thin polyester mat with an acrylate coating. The material allows adaption to the ambient air temperature almost instantaneously and, thus, serves as a proxy for the local air temperature (Grudzielanek and Cermak 2015). As the screens are not produced for scientific purposes, the material shows imperfections such as thickness variations. Furthermore, it is sensitive to contamination with dirt, which changes the reflectivity and the heat capacity. The screens are spanned with an elastic rope in a metal frame to create a trampoline-like set-up, which ensures that the metal structure has a minimal effect on the flow field and the screen temperatures.

A two-dimensional ultrasonic anemometer (WindSonic Gill Instruments, Lymington, UK) is installed on the downwind side of the set-up, (‘3’ in Fig. 2a) mainly providing information about wind direction. These data are used to select time periods where the wind direction is aligned with the screen axis to avoid an influence of the screens on the flow field. For wind directions deviating more than $30^\circ$ from this axis, substantial flow field disturbances such as up- or downdrafts can be observed. Furthermore, a portable short-path three-dimensional ultrasonic anemometer, deployed close to the surface in vicinity of the screens, provides measurements for the validation of screen data.

### Wind Field Approximation

The high spatio-temporal resolution of the TIR camera allows for the investigation of boundary-layer dynamics. The air temperature of a plume is used as a marker to observe its motion. The wind field estimation from infrared data (WEIRD) method tracks the pattern caused by air temperature gradients moving over time and estimates a two-dimensional wind field from this. Heterogeneities are induced by the strong differences in surface temperatures between bare ground and snow and the advection of warm air from snow free towards snow covered areas. The resulting air temperature gradients in both the horizontal and the vertical direction allow an approximation of the horizontal and vertical velocity components. Stronger heterogeneities in air temperature yield more reliable estimates of the wind field. The two-dimensional wind field is a projection of the real three-dimensional wind field onto the plane of the screens. A validation of the estimated wind fields is presented in Sect. 3.3.

**Fig. 3:**
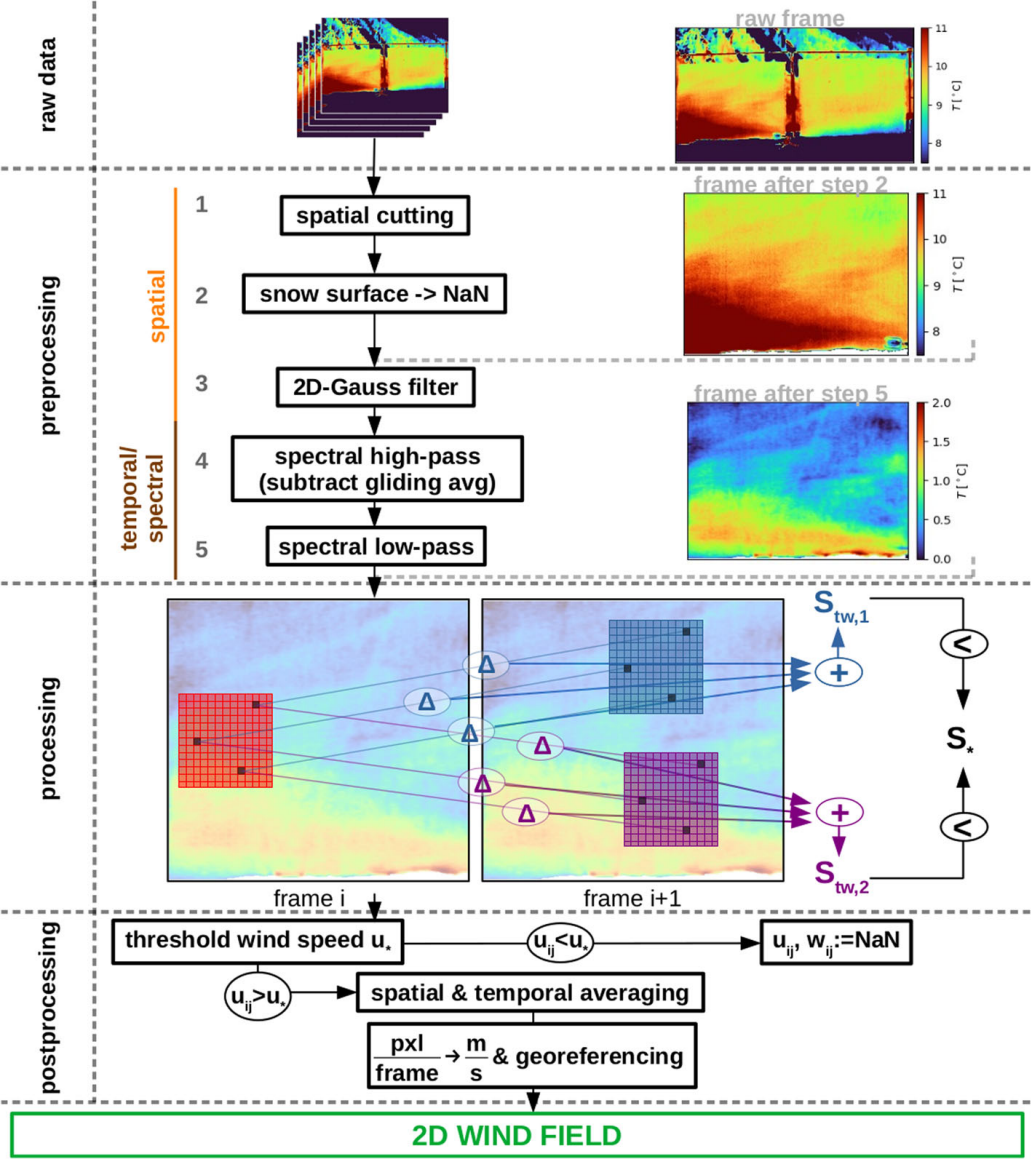
Sketch of the WEIRD method: The raw infrared sequence is preprocessed in several steps including spatial and temporal filters. In the processing step, the correlation between an interrogation window in frame *i* (red) and multiple spatially shifted target windows in the subsequent frame $i+1$ (blue, purple as two examples) is calculated. The spatial shift between the interrogation window and the target window with the highest correlation is used to calculate the wind speed. The postprocessing includes the application of a threshold wind speed and averaging depending on the desired properties of the two-dimensional wind field. For further details, see text

The WEIRD method consists of different preprocessing, processing, and postprocessing steps. The steps are shown in the overview sketch in Fig. 3 and explained in detail in the following. Documented source code can be found online (see Sect. 4).

#### Preprocessing

The goal of the preprocessing is to reduce the noise contained in the infrared frames in order to improve the approximation of the wind speed. The noise mainly stems from imperfections of the screens as well as from artefact(s) caused by the microbolometer (see Sect. 2.1.1). In the preprocessing, the raw infrared data are filtered in both the spatial and the spectral domains. The sequence of preprocessing steps is shown in the upper part of Fig. 3. On the right, an example frame is shown as raw data (top), after preprocessing step 2 (middle), and after complete preprocessing (bottom).

The first three preprocessing steps are applied to all infrared frames independently and tackle the spatially distributed noise. Steps 4 and 5 act on the temperature time-series of each pixel and, thus, dampen the temporal or spectral noise. In the following, the single steps are described in more detail:

1. The raw infrared sequence is first spatially cut to remove the surrounding environment. Thereby the number of pixels per frame is reduced by $60\%$ and, thus, sufficiently saves computation time for all following steps.
2. Data points representing the snow surface below the screens are set to not-a-number (‘NaN’) by applying a temperature threshold to every pixel in the lower part of the infrared frame. The value of this threshold depends on the temperature offset of the TIR camera and the angle of view (Pestana et al. 2019) and is set individually for an infrared sequence. It ensures that the wind field can also be evaluated close to the snow surface without artificial influences of the snow surface. In Fig. 3, an example of an excerpt from a frame after preprocessing steps 1 and 2 is given on the right side. In contrast to the same frame from the raw infrared sequence shown above, it is cut to screen dimensions and the snow surface is set to NaN (shown by white colour).
3. *Spatially anisotropic Gauss filtering* dampens noise in the spatial domain by smoothing the pixel values. It is applied frame-wise to reduce the vertically aligned and temporally varying artefact(s). The anisotropy of the noise is assumed to originate from the column-wise read-out of the microbolometer. To account for the vertical structure, the smoothing over columns (lateral) is stronger than over rows (vertical), i.e. $\sigma _{\textrm{col}}=2$ pixels and $\sigma _{\textrm{row}}=1$ pixels.
4. The *subtraction of a back-looking time average* removes effects of low frequency changes. Possible sources include effects caused by the TIR camera such as the temporally changing vignetting. Furthermore, small particles of dirt with different reflectivity and heat capacity than the screens show a different response to incoming shortwave radiation. These effects are dampened by subtracting a back-looking 10-s moving time average similar to a spectral high-pass filter. The resulting data represent temperature fluctuations instead of absolute values.
5. Finally, a *spectral low-pass filter* is applied to each pixel time series. A frequency analysis of the measured temperature data (see Online Resource 1) points to the fact that the screens react to air temperature changes on a time scale of approximately 1 s. Faster changes are strongly damped due to the heat capacity of the screen material and superimposed by random noise. To minimize these influences, a cut-off frequency of $f_{co}=2\,\textrm{Hz}$ is chosen for the spectral low-pass filter. The bottom infrared frame on the right side of the preprocessing part in Fig. 3 shows the frame after complete preprocessing. Note that the colour bar shows temperature fluctuations $T'$, due to the subtraction of a gliding average in step 4. Also note that the temperature range is $2.0\,^\circ \hbox {C}$ in contrast to $3.5\,^\circ \hbox {C}$ in both the raw infrared frame and the infrared frame after step 2. The column-wise noise from the previous excerpt is averaged out. However, there is a diagonal line throughout the height of the screen visible. It originates from thickness variations of the screen, probably caused in the manufacturing process.

#### Processing: Wind Field Estimation Using Thermal Infrared Data

The WEIRD processing step leads to a raw approximation of the two-dimensional wind field in the predefined spatial excerpt of the infrared sequence. Therefore, pairs of two subsequent frames of the preprocessed sequence are analysed. In the following, they are denoted by frame *i* and frame $i+1$. The time interval between those two frames is given by the temporal resolution of the TIR camera $\varDelta t=\frac{1}{{30}\,{Hz}}\approx {3\times 10^{-2}}\,\hbox {s}$. Vertical velocity component *w* is defined to be perpendicular, whereas horizontal wind speed *u* is parallel to the bottom of the infrared frame.

For the following best-correlation-search, both frames are spatially subdivided into windows which are called *interrogation windows* in frame *i* and *target windows* in frame $i+1$. Because neighbouring windows are shifted by one pixel, they largely overlap. The size of those windows depends on the structure of the observed pattern and, thus, on the structure and dimensions of the investigated near-surface atmospheric layer processes. For our measurement set-up, a parameter study yielded an optimal window size of $10 \times 10~\textrm{pixels}$ (not shown). In Fig. 3, interrogation and target windows are shown within the processing part. The red rectangle in frame *i* denotes an interrogation window. Similarly, the blue and purple rectangles in the right frame symbolize two (out of 300 per interrogation window as in this study) target windows. The grid symbolizes the pixels in this window. To estimate the wind field, correlations between one interrogation window and a set of spatially shifted target windows are calculated. Therefore, values for the maximum shift in positive and negative horizontal and vertical direction need to be defined. These values define the *search region* in frame $i+1$ containing all analysed target windows for a given interrogation window. For this study, the search region is estimated using ultrasonic wind measurements (see Sect. 2.3). It is set to be symmetric in vertical direction ($-5$ pixels to $+5$ pixels) accounting for a symmetric distribution of the measured vertical wind speeds and with a bias ($-5$ pixels to 25 pixels) in the direction of the measured horizontal wind speed in horizontal direction. The search region also defines the extent of the estimated wind field towards the upwind, downwind, and top edges of the screen. Only interrogation windows for which all target windows are within the screen extent are considered. Since information on the wind field is of special interest close to the snow surface, the vertical extent of the search region decreases symmetrically towards the surface in the lowest $0.1\,\hbox {m}$ of the screen. This is in accordance with decreasing vertical velocity component close to the land surface. However, no decrease in the horizontal extent of the search region close to the bottom is applied to allow the detection of low-level jets (Mott et al. 2020).

The wind field approximation for two screens and one time step needs $\mathcal {O}(10^{8})$ correlations to be evaluated. Therefore, a computationally efficient algorithm to estimate correlations is adapted from Kaga et al. (1992). This algorithm finds the best correlation between a given interrogation window and one out of the set of target windows by accumulating differences between corresponding pixels step by step for each target window. Once the sum exceeds a defined threshold, the respective target window is not considered anymore. The last remaining target window is assumed to have the highest correlation with the interrogation window. This search is done in three steps (Kaga et al. 1992):

1. The accumulated absolute difference $S_{tw, k}$ between $n_{\textrm{ini}}=10$ random pixels in the interrogation window and all target windows *k* is evaluated. $S_{tw, \textrm{min}}$ denotes the minimum of all $S_{tw,k}$ values.
2. The threshold $S_{*}$ for aborting the correlation search is set as:
  2 $$\begin{aligned} S_{*}=\alpha S_{tw}\cdot \frac{x_{w}}{n_{\textrm{ini}}}, \end{aligned}$$
  with $\alpha$ a constant and $x_w=10$ the window size. Lower values of $\alpha$ lead to a faster correlation search, but may also lead to a non-unique determination of the best correlation. Sensitivity testing showed that $\alpha =0.3$ gives a good balance.
3. The summation is continued with step-wise accumulation of differences between random pairs of pixels. Once $S_{tw,k}>S_{*}$, the corresponding target window *k* is not considered anymore. The last remaining target window is assumed to have the highest correlation with the interrogation window. This step is illustrated in Fig. 3.

Often a unique best-correlation target window cannot be determined using this algorithm. This can happen when multiple target windows exceed the threshold within one step of the pixel-wise difference and no target window with $S_{tw,k}<S_{*}$ is left. Thus, an additional method to compare these multiple qualified target windows is needed. Therefore, not only the number of steps until $S_{tw,k}>S_{*}$ (which is the same for the remaining target windows in this case), but also $S_{tw,k}$ directly is used as a secondary criterion. Applying this correlation search for all possible interrogation windows and for all pairs of subsequent infrared frames in a sequence yields a two-dimensional wind field and its temporal evolution.

#### Postprocessing

The WEIRD method is not able to estimate a wind field within a moving plume of constant air temperature. When this occurs, WEIRD may calculate erroneously low wind speed estimates due to a lack of temperature inhomogeneities. Therefore, the first step in the postprocessing is to apply a threshold $u_* = +1~\mathrm {pixel(s)}~\textrm{frame}^{-1}$ to the horizontal velocity component. If the estimated horizontal velocity component is below this threshold, the horizontal and vertical velocity components at this pixel is set to NaN.

In order to smooth the resulting two-dimensional wind field, averaging in both the spatial and the temporal domain can be applied. The balance between spatial and temporal averaging depends on the expected properties of the wind field. For example, if the focus is on a high temporal resolution of the estimated wind field, averaging over a domain with larger spatial but smaller temporal extent might be applied. The rate of NaNs within an averaging window (in both spatial and temporal dimension) is tracked and used as a quality control measure.

In a final step, the obtained shift in $\mathrm {pixel(s)}~\textrm{frame}^{-1}$ is converted into wind speed in $\hbox {m}\,\hbox {s}^{-1}$. With the spatial resolution in horizontal direction $\varDelta _x = 0.61\,\hbox {cm}$ and the frame rate of the TIR camera $f=30\,\hbox {Hz}$, the conversion factor for horizontal wind speed *u* is given by $\varDelta _x \cdot f = 0.183\ \frac{\textrm{m} \cdot \textrm{frame}}{\textrm{s} \cdot \textrm{pixels}}$. Similarly, the conversion factor for vertical velocity component *w* is $\varDelta _z \cdot f = 0.55\ \frac{\textrm{cm}}{\textrm{pixels}}\cdot 30\,\hbox {Hz} = 0.165 \frac{\textrm{m} \cdot \textrm{frame}}{\textrm{s} \cdot \textrm{pixels}}$. Be aware that the raw estimated wind speeds for a single pixel and two subsequent frames can only be an integer multiple of the conversion; factors $u_{\textrm{min}}=0.183\,\hbox {m}\,\hbox {s}^{-1}$ and $v_{\textrm{min}}=0.165\,\hbox {m}\,\hbox {s}^{-1}$ are the minimal detectable wind. A quasi-continuous wind speed estimation is achieved by temporal and spatial averaging of single pixel estimations. The two-dimensional wind vector is assigned to the centre of the corresponding interrogation window. Each vector’s location in spatial coordinates (fetch distance and height) is obtained by georeferencing.

### Turbulence Measurements

In addition to the screens, a portable short-path ultrasonic anemometer (DA-700 Sonic Corporation, Tokyo, Japan; in the following ‘turbulence sensor’) was deployed at a height of $h_{K}=0.35\,\hbox {m}$ above the surface. With the measured three-dimensional velocity components *u*, *v*, *w* and the virtual potential temperature $\theta$, the turbulent vertical sensible heat fluxes (SHFs) can be calculated. Data from the turbulence sensor are evaluated for the same 10 min as the infrared sequence. Prior to flux computation, erroneous data points are removed and wind data are detrended (Aubinet et al. 2012). Furthermore, the three-dimensional velocity data are double rotated (Kaimal and Finnigan 1994). Double rotation is preferred over planar fitting due to permanent surface changes induced by snow melt and slight movement of the sensor (Stiperski and Rotach 2016). After rotation of the ultrasonic data, the *u*-component points into the mean wind direction and the *w*-component is perpendicular to the snow surface (Mott et al. 2020). In order to define a suitable Reynolds averaging time separating turbulent from non-turbulent (sub)mesoscale motions, data from the turbulence sensor are evaluated using a Multi-Resolution Flux Decomposition (MRD), which is a wavelet transformation preserving Reynolds averaging rules (Vickers and Mahrt 2003). However, due to the strong temporal heterogeneity of the near-surface atmospheric layer, it is challenging to determine a clear gap scale between turbulent and (sub-)mesoscale motion as described in Vickers and Mahrt (2003). Combining the MRD with sensitivity analysis suggests a gap scale of $t_{\textrm{avg}}=30\,\hbox {s}$. Reynolds averaging to compute fluxes is applied using a moving average with a symmetric window of width $t_{\textrm{avg}}$ around every data point. The obtained SHFs are smoothed by a moving average with a width of 10 s. Additionally, data measured by the turbulence sensor are used as a validation measurement for the wind speed estimations (see Sect. 3.3).

## Results and Discussion

In the following, an infrared frame is examined qualitatively to obtain a snapshot of the near-surface atmospheric layer. Subsequently, vertical profiles of horizontal and vertical velocity and temperature for the same situation are analysed to demonstrate the ability of the developed method. Measurements of velocity and calculated sensible heat fluxes close to the screen allow the validation of the WEIRD wind field estimations. A video showing the evolution of the near-surface atmosphere in real time is included in Online Resource 2.

### Near-Surface Atmospheric Layer Structure Information Gained from an Infrared Frame

**Fig. 4:**
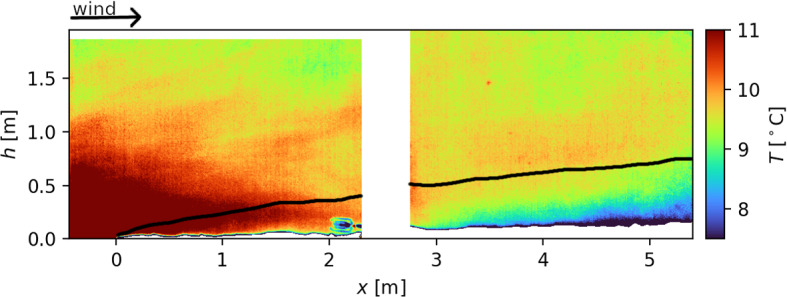
A frame out of the sequence for qualitative analysis. The wind advects warm air above bare ground (left/upwind of $x=0\,\hbox {m}$) over the snow surface. The black line visualizes the internal boundary-layer-height approximation in Granger et al. (2006)

Qualitative analyses of the data from the TIR camera reveal information about the structure of the near-surface atmospheric layer. Figure 4 shows one infrared frame as an excerpt from the infrared sequence with the arrow indicating the prevailing wind direction. The figure shows the wind fetch distance over snow, *x*, on the *x*-axis and the height above the bottom of the infrared frame on the y-axis. The snow cover at the screen location is shown in Fig. 2a. Furthermore, the UAV image in Fig. 1 gives an overview of the snow cover fraction on the site. According to preprocessing step 2, snow covered pixels in Fig. 4 are set to NaN and are shown in white. The pixels between the two screens are set to NaN since they do not contain information on the near-surface dynamics. At $x_K=2\,\hbox {m}$, the turbulence sensor is deployed at a height of $h_K=0.35\,\hbox {m}$ above the snow surface (visible in the foreground of Fig. 4, 1.5 m in front of the screens). Data measured with the turbulence sensor are used for validation of the estimated wind field in Sect. 3.3. Furthermore, at $x=3.5\,\hbox {m}$ and a height of $1.5\,\hbox {m}$ above the bottom of the frame, a small area with a warmer screen temperature is visible. This is an artefact stemming from dirt with a reduced reflectivity and increased heat capacity compared to the screen material. The isolated artefact has just local influences on the recorded temperature field and, subsequently, also on the estimated wind field. However, it demonstrates the importance of a clean screen surface.

The enhanced surface temperature and upward heat fluxes at the bare ground induce plumes of warm air. Figure 4 shows the advection of a warm air plume with the mean wind towards the snow covered area. Adjacent to the snow surface a centimetre-deep film of cold air is visible along the whole screen length. There is a strong air temperature gradient within this small film from $0\,^\circ \textrm{C}$ at the snow surface to $\approx 10\,^\circ \textrm{C}$ above. In order to better visualize the temperature differences above this thin film, the lower limit of the colour bar is chosen to be $7.5\,^\circ \textrm{C}$. Since the temperatures in the lowest centimetre above the snow surface are below this lower limit, the occurring temperature gradient is not resolved in Fig. 4 and the temperature appears to be constant within the centimetre adjacent to the snow surface. Above, for $x<0.5\,\hbox {m}$ the warm air is mixed down to the snow surface. However, for $x>0.5\,\hbox {m}$ a layer of colder air adjacent to the snow surface is visible. With increasing fetch distance, the depth of the cold air layer increases. Simultaneously, the air temperature close to the snow surface decreases with increasing fetch distance. This layer of cold air yields a statically stable temperature stratification close to the ground (temperature profiles are discussed in more detail in Sect. 3.2). Above the cold air layer, the static stability changes. The transition in static stability might be referred to as the stable internal boundary layer (IBL) height. Internal boundary layers are induced by air moving across a transition of different surface properties (Garratt 1990). In our case, there is a step change in surface temperatures from bare ground (warm) to snow covered areas ($0\,^\circ \textrm{C}$). Additionally, there is a step change in surface roughness. In an experimental study, Granger et al. (2006) measured the height of the IBL at various fetch distances over snow on an isolated snow patch. The black line in Fig. 4 shows their best-fit exponential function as an approximation for the IBL growth for an upwind surface roughness of $z_0=0.02\,\hbox {m}-0.04\,\hbox {m}$, which is comparable with the surface roughness of the alpine meadow in our case. In contrast to point-wise temperature measurements as used, for example, in Granger et al. (2006) and Harder et al. (2017), the high spatio-temporal resolution of the temperature field in Fig. 4 facilitates more detailed spatial and temporal investigations. Comparing approximated IBL growth with the temperature field on the downwind (right) screen reveals an analogous behaviour especially for the downwind screen. For increasing fetch distances, the height at which a given temperature level is reached grows similarly (e.g. cyan colour $T\approx 9\,^\circ \textrm{C}$). This resemblance affirms the theoretical approximation. However, a clear IBL height is difficult to determine only from the temperature field. Also note that Fig. 4 shows a snapshot of the temperature stratification. For $x<1.5\,\hbox {m}$, significant penetration of warm air below the black line down to the snow surface can be observed. Warm air is mixed into the stable IBL at the upwind edge of the snow patch. This entrainment leads to increased melt rates at the upwind edge of the snow patch and is referred to as the leading edge effect. Besides the asset of spatially continuous temperature fields at centimetre resolution, the high temporal resolution of data recorded with the TIR camera allows us to examine the evolution and dynamics of the near-surface atmospheric layer.

### Vertical Profiles of Temperature and Estimated Wind Speeds

**Fig. 5:**
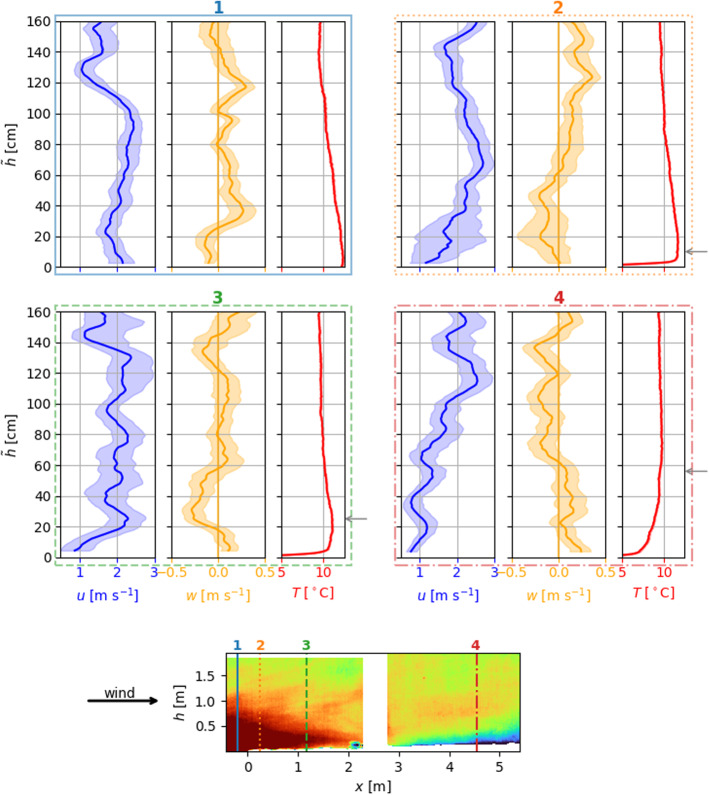
Vertical profiles of horizontal velocity component *u* (blue), vertical velocity component *w* (orange) and temperature *T* (red) for 4 different locations along the screen axis averaged over $0.25\,\hbox {s}$. ${\tilde{h}}$ is the height above the snow surface for locations 2–4 and the height above bare ground for location 1. $w=0\,\hbox {m}\,\hbox {s}^{-1}$ is marked by an orange solid vertical line. The four locations are indicated by the vertical lines in the bottom infrared frame and the frames around the corresponding profiles. The height *h* in the infrared frame refers to the height above the bottom of the frame. The top left profiles describe the situation above the upwind bare ground. From the top right profiles to the bottom right profiles, the fetch distance over snow increases. Therein, the approximation for the SIBL height by Granger et al. (2006) is indicated by the grey arrows on the right

Figure 5 shows vertical profiles at four locations along the screens (1 to 4). The profiles describe the near-surface atmospheric layer dynamics as measured in Fig. 4. Complementary to the measured static boundary-layer information shown in Fig. 4, the profiles reveal additional high spatial resolution estimations about the dynamics close to the surfaces retrieved by the WEIRD method. Vertical profiles provide information on horizontal wind speed *u* (left, blue), vertical wind speed *w* (middle, orange), and screen temperature *T* (right, red) as a proxy for air temperature. The vertical velocity component *w* is defined to be positive if air moves upwards away from the surface. The vertical line in the *w*-profiles denotes $w=0 \textrm{m}\,\textrm{s}^{(-1)}$. Be aware that, as shown in the bottom infrared frame, the height of the snow surface (${\tilde{h}}=0\,\textrm{cm}$) increases for increasing fetches. The vertical lines in the infrared frame indicate the profiles’ location on the screens and the corresponding fetch distance. Profile 1 shows the near-surface atmospheric layer over bare ground upwind of the snow patch, while 2 and 3 are taken at fetch distances of $x_2=0.25\,\hbox {m}$ and $x_3=1.15\,\hbox {m}$. The vertical structure of the near-surface atmospheric layer further downwind at a fetch distance of $x_4=4.60\,\hbox {m}$ is indicated by profile 4.

In order to obtain the vertical profiles as shown in Fig. 5, the wind field is averaged using a pixel-wise 0.5 s moving average (corresponding to 15 frames) as described in Sect. 2.2.3. The solid lines in the profile plots show the median of all raw profiles in an averaging window with spatial width of $\varDelta x = {10}\,\hbox {cm}$ and a temporal span of $\varDelta t = 0.25\,\hbox {s}$. The shaded regions indicate the interquartile range containing $50\%$ of the data from the raw profiles around the median. For a smoother visualization, the profiles are averaged vertically by a moving average with an averaging interval length of $15\,\hbox {cm}$. Comparing the temperature profiles from the four fetch distances 1 to 4 shows the cooling of the near-surface air as it is transported over the snow surface. This effect is visible up to a height of $\tilde{h}=h_B\approx 100\,\hbox {cm}$. Vertical temperature profiles measured by Harder et al. (2017) and idealized direct numerical simulations by van der Valk et al. (2021) reveal a similar behaviour. Harder et al. (2017) refer to $h_B$ as the blending height. Furthermore, the temperature profiles 1 to 4 visualize the changing static stability of the near-surface atmospheric layer across the transition from bare ground to snow surface as mentioned in Sect. 3.1. The air over bare ground at location 1 is characterized by a statically unstable stratification below a height of $\approx 120\,\hbox {cm}$ and a near-neutral stratification above. This static instability stems from the bare ground heating up the adjacent air mass causing plumes of warm air. At the leading edge of the snow patch in profiles 2, a very shallow statically stable layer close to the snow surface up to $\tilde{h} \approx 5\,\hbox {cm}$ is visible. Further downwind, the thickness of the statically stable layer close to the snow surface grows. For all locations, the IBL height approximated by Granger et al. (2006) is indicated by the grey arrows on the right of the profiles. Comparing the approximation and the height of the statically stable layers at locations 2–4 displays a similar growth. A growing stable layer adjacent to the snow surface is also in accordance with the findings in van der Valk et al. (2021). They show that the strongest downward SHF occur at the upwind edge of the snow patch and the magnitude of the SHF decreases with increasing fetch distance due to a growing stable layer. However, these profiles and the static stratification at the different locations are highly variable in time.

As mentioned above, the entrainment at the leading edge of the snow patch is characterized by mixing warmer air from above down into the IBL at the snow surface. Negative vertical velocity components for $h<60\,\hbox {cm}$ at location 2 and for $20\,\hbox {cm}<h<60\,\hbox {cm}$ at location 3 point to this downward mixing.

**Fig. 6:**
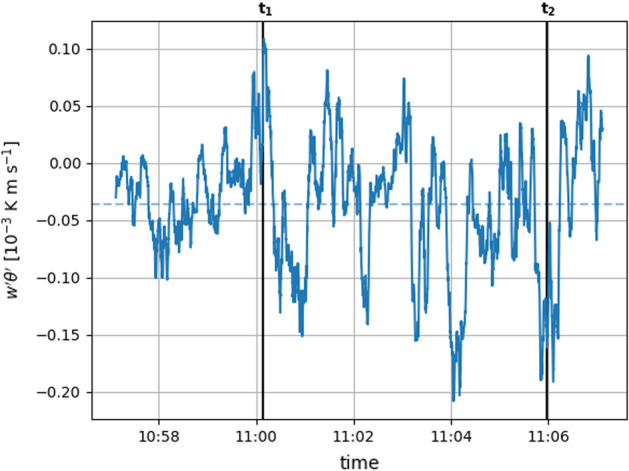
Kinematic turbulent vertical sensible heat fluxes ($w'\theta '$, SHFs) measured by the short-path ultrasonic (turbulence sensor) at $h_K=0.35\,\hbox {m}$ and a fetch distance $x_K=2\,\hbox {m}$ (see bottom right of the upwind screen in Fig. 4). Fluctuations of vertical velocity component $w'$ and temperature $\theta '$ result from subtracting the 30 s mean from the time series. The plotted flux is averaged using a 10 s moving average. Positive (negative) SHFs refer to the transport of heat away (towards) the surface. The dashed horizontal line represents the average. The vertical line ‘$\textrm{t}_1$’ (positive SHFs) shows the time where the profiles in Fig. 5 and the left column of Fig. 7 are taken. The vertical line ‘$\textrm{t}_2$’ (negative SHFs) indicates the time for the profiles and the frame in the right column of Fig. 7

At location 2 below $40\,\hbox {cm}$, an enhanced spread in the horizontal and vertical velocity components is visible through the wider interquartile range. This spread points to the extreme spatio-temporal variability of the flow directly at the leading edge of the snow patch. Another aspect is the reduced horizontal velocity component *u* in the statically stable layers at locations 3 and 4 in comparison to *u* aloft. Furthermore, the change in static stability is accompanied by a change from positive *w* in the statically stable regime to negative *w* above. These changes might point to a decoupling of the statically stable layer adjacent to the snow surface from the atmosphere above. At location 2, the statically stable layer is too shallow, so a change of vertical velocity component direction cannot be resolved. However, a reduction in *u* close to the surface can be observed. These findings are complemented by vertical turbulent sensible heat fluxes calculated from the DA-700 short-path ultrasonic (turbulence sensor) data. Figure 6 shows a time series of the kinematic SHFs at the same time interval as the infrared sequence.

Be aware that the ultrasonic and the infrared data are not precisely synchronized so there might be a time shift in the order of a few seconds. The calculated SHFs vary strongly between up- and downward. This heterogeneity of the fluxes suggests that the turbulence sensor is close to the height of the temporally varying stable IBL, which is in accordance with the approximation in Granger et al. (2006) and to the profiles in Fig. 5. Although strongly oscillating and changing signs, the SHFs averaged over the whole sequence are slightly negative (dashed horizontal line), indicating a mean IBL height just above the measurement height of the turbulence sensor.

**Fig. 7:**
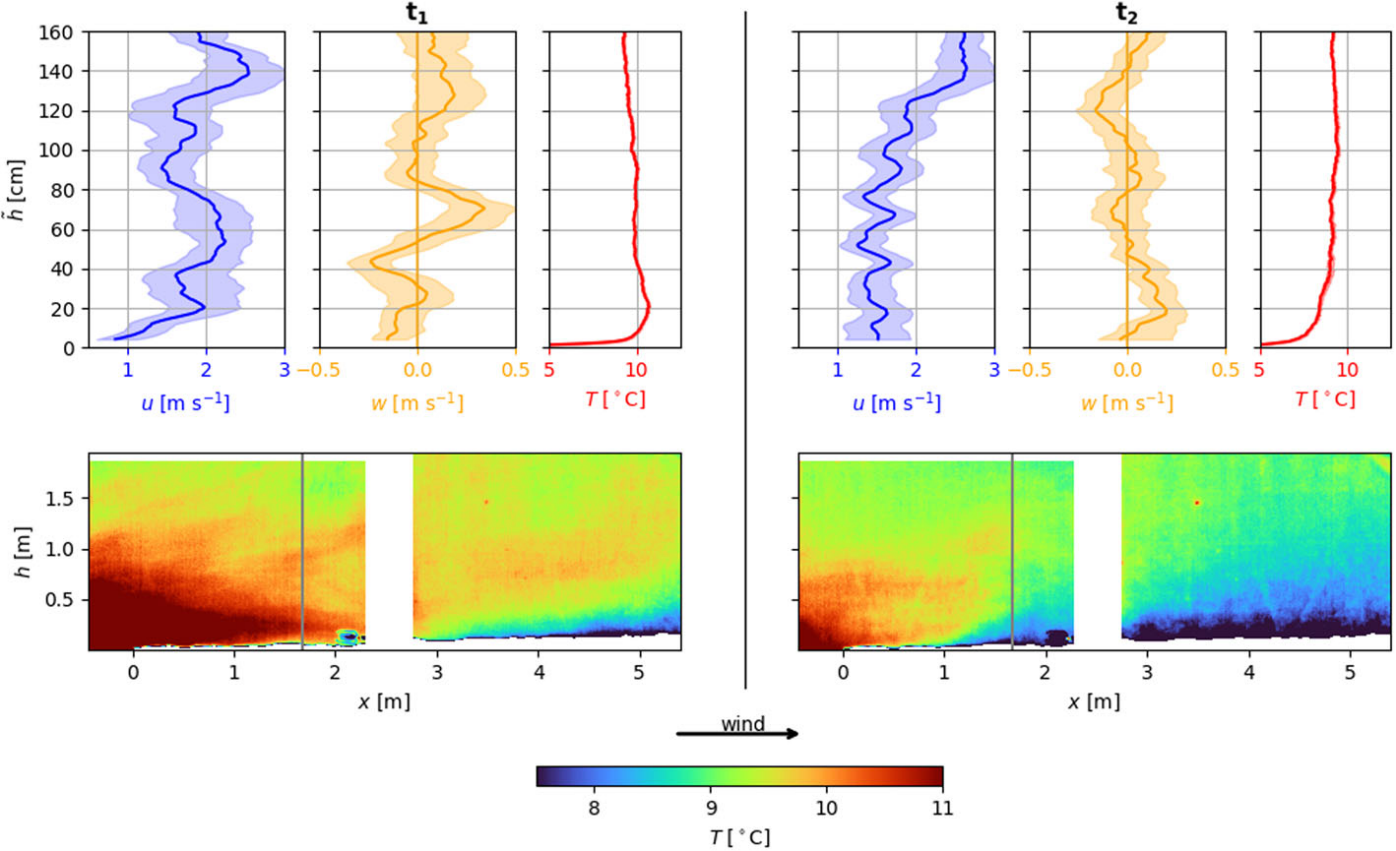
Juxtaposition of profiles and infrared frames for positive SHFs ($\textrm{t}_1$) and negative SHFs ($\textrm{t}_2$) at the height of the turbulence sensor. See the vertical lines in Fig. 6 for the measured fluxes

In order to compare two different characteristic situations, Fig. 7 juxtaposes profiles and an infrared frame for a time when the turbulence sensor registered positive SHFs (vertical line $\textrm{t}_1$ in Fig. 4) in the left column and negative SHFs (vertical line $\textrm{t}_2$ in Fig. 4) in the right column.

Time $\textrm{t}_1$ represents the same situation as shown in Figs. 4 and 5. However, the profiles are taken at a fetch distance comparable to the fetch distance of the turbulence sensor as indicated by the vertical line in the infrared frame. Comparing the infrared frames of time $\textrm{t}_1$ and $\textrm{t}_2$ reveals a significantly different atmospheric structure. The atmosphere at time $\textrm{t}_1$ is characterized by entrainment of warm air at the leading edge of the snow patch and a thin layer of cold air adjacent to the snow surface further downwind. At time $\textrm{t}_2$, the air is generally colder and the layer of cold air is thicker. At a height of $\tilde{h}= 20\,\hbox {cm}$ above the snow surface, the temperature difference between $\textrm{t}_1$ and $\textrm{t}_2$ is $2.2\,^\circ \hbox {C}$. Warm air from over bare ground is not moving downwards towards the snow surface. The thicker and colder layer of air can also be seen in the temperature profiles. While the stable layer at time $\textrm{t}_1$ is only $20\,\hbox {cm}$ deep, its depth is ${100}\,\hbox {cm}$ at time $\textrm{t}_2$. In both cases, the horizontal velocity component in the stable layer is lower than above. At time $\textrm{t}_1$ and above the warm air plume, a transition from slightly negative vertical velocity component to positive vertical motion can be observed. Within the stable layer at time $\textrm{t}_2$, updrafts occur close to the surface. Above $\tilde{h}>0.5\,\hbox {m}$, the vertical motion is very weak. Combining the infrared frames and the profiles, the findings are in accordance with the SHFs calculated from the turbulence sensor data. For time $\textrm{t}_1$, positive SHFs indicate a very shallow IBL below the measurement height of the turbulence sensor, whereas for time $\textrm{t}_2$, negative SHFs support the increased depth of the IBL. The fact that both situations were observed within few minutes demonstrates the high temporal variability of the atmosphere adjacent to the snow surface and therefore, the need for ultra-high resolution measurements.

### Validation Using Wind Measurements

**Fig. 8:**
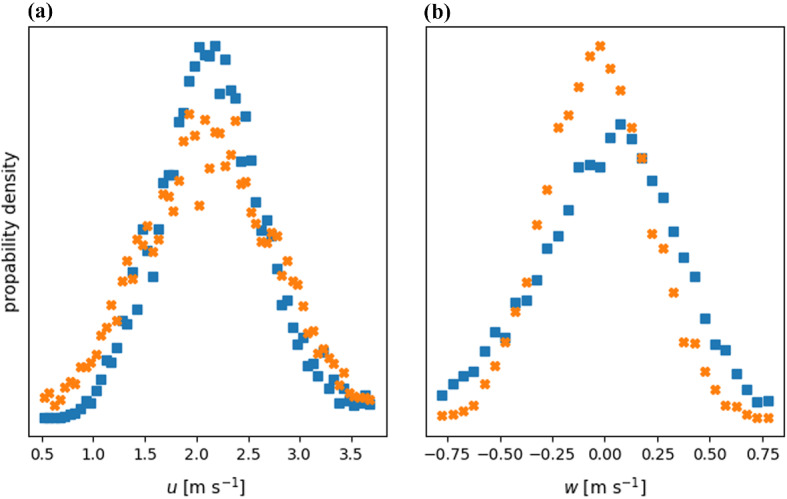
Histogram comparison between measured ultrasonic data (turbulence sensor, blue) and estimations using WEIRD (orange) for **a** horizontal and **b** vertical wind speed components

For the validation of the near-surface wind field estimations from WEIRD, measured wind speeds from the short-path ultrasonic anemometer (turbulence sensor) are used. Data recorded with the turbulence sensor are double rotated into the mean wind speed. To overcome the differences in temporal resolution of the turbulence sensor ($20\,\hbox {Hz}$) and WEIRD estimations (raw data $30\,\hbox {Hz}$), both are averaged to 10 Hz. Furthermore, ensuring comparability between the two data series, the WEIRD estimations are spatially averaged over a $5 \times 5\,\hbox {cm}^{2}$ window, which matches the path length of the turbulence sensor. The location of the WEIRD averaging window is chosen at the same fetch distance as the profiles in Fig. 7 at a height of $h=h_K=0.35\,\hbox {m}$. Due to the strong spatio-temporal heterogeneity of near-surface turbulence, a comparison of the wind speed distributions over the $10\,\hbox {min}$ observation period of the infrared sequence is adequate. The distributions are obtained by binning both time series using a bin with of $0.05\,\hbox {m}\,\hbox {s}^{-1}$. Figure 8a shows the comparison for the horizontal velocity component *u* and Fig. 8b for the vertical component *w*.

The comparison of horizontal wind speed histograms in Fig. 8a yields a correlation of $\rho _u=0.96$ and a root-mean-square deviation of $RMSD_u = 0.10$. Both the turbulence sensor data and the WEIRD estimations show a similar distribution with a peak at $\hat{u}\approx 2.2\,\hbox {m}\,\hbox {s}^{-1}$. However, the maximum of the WEIRD distribution exhibits more scatter. Furthermore, WEIRD estimations of $u<1.5\,\hbox {m}\,\hbox {s}^{-1}$ occur more often than for the turbulence measurements. A possible explanation might be the decreased performance of the estimation for homogeneous air temperatures or very rapid changes between two subsequent infrared frames leading to difficulties in tracking the pattern. As a consequence, the WEIRD algorithm yields wind speeds scattered around $0~\text {pxl frame}^{-1}$. This scatter is partly filtered by applying the threshold wind speed $u^*=+1~\text {pxl frame}^{-1}$, but the remaining scatter overlaps with valid estimations resulting in an increased estimation of $u<1.5\,\hbox {m}\,\hbox {s}^{-1}$. Additionally, an increased occurrence of estimations for $u>2.8\,\hbox {m}\,\hbox {s}^{-1}$ can be observed. This might stem from the fact that high horizontal velocity components in the experimental setting of this study mainly coincide with the advection of a warm air plume and, thus, an increase in local air temperature. The resulting clear air temperature pattern allows for accurate tracking and consequently for a valid wind estimation. However, if the near-surface flow is characterized by a homogeneous air temperature over time, tracking of pattern fails and results in the above described scatter. The resulting withdrawn estimations leads to a relative overestimation of high (and valid) estimations.

The distributions for the vertical velocity component *w* shown in Fig. 8b exhibit a correlation of $\rho _w = 0.94$ and a root-mean-square deviation of $\textrm{RMSD}_w = 0.23$. Both distributions show a similar shape, but with a shifted peak. This shift might stem from a small tilt of the local snow surface. This tilt would result in a tilt between the direction of horizontal velocity component ($w_{\textrm{WEIRD}}=0\textrm{m}\,\textrm{s}^{(-1)}$) on the screen and the *u*, *v*-plane of the turbulence sensor. The applied double rotation on the turbulence sensor data implies $\overline{w}_{\textrm{turbsensor}}=0\textrm{m}\,\textrm{s}^{(-1)}$, whereas $w_{\textrm{WEIRD}}:=0\textrm{m}\,\textrm{s}^{(-1)}$ is defined for motions parallel to the bottom of the infrared frame. A counter clockwise rotation of the local wind field in the chosen average window of $\beta = 0.97^\circ$ would yield $\overline{w}_{\textrm{WEIRD}}=0\textrm{m}\,\textrm{s}^{(-1)}$ and could therefore explain the shift. However, the minimal shift towards negative vertical motions estimated with WEIRD might also represent the true motion of air, which might have been removed from the turbulence sensor data through double rotation. Similar to the analysis of the horizontal velocity components, the higher occurrence of WEIRD estimations around $w=0\,\hbox {m}\,\hbox {s}^{-1}$ can be explained by scatter around $w=0\,\hbox {m}\,\hbox {s}^{-1}$, where the algorithm cannot track a clear air temperature pattern. This scatter, and the resulting increased probability density around $w=0\,\hbox {m}\,\hbox {s}^{-1}$, might also be a reason for the relative underestimation of stronger positive and negative vertical motions.

Generally, the wind field estimations using the WEIRD algorithm could be improved by including the propagation of information from the wind field estimation between frame *i* and frame $i+1$ to the subsequent step. This could help to significantly reduce the computation time due to a restriction of possible target windows to a smaller spatial domain. Additionally, propagation of information between subsequent steps, especially limiting the number of possible target windows, may yield a smoother wind field after the processing step. A smoother wind field in turn offers the possibility to retrieve even higher spatio-temporal resolution data, since less averaging in the post processing is necessary.

## Conclusion and Outlook

We presented a method to gain information about the near-surface atmospheric layer and its dynamics over a strongly heterogeneous surface. Thin synthetic screens were set up vertically across a transition from bare ground to snow aligned with the predominant wind direction. Combined with infrared measurements, the screens serve as a proxy for air temperature. Sequences of infrared frames are recorded by a high-resolution thermal infrared camera at 30 Hz. These data yield ultra-high spatio-temporal resolution information about the near-surface air temperature stratification. Various processes could be observed, such as the entrainment of warm air at the leading edge of a snow patch, or the development of a stable internal boundary layer adjacent to the snow surface.

Using temperature as a marker for moving air parcels, the WEIRD method can track temperature patterns and estimate high-resolution horizontal and vertical velocity components. In combination with the temperature stratification and its temporal change, the wind field from WEIRD provides a new way to quantify the dynamics of the near-surface atmospheric layer on scales of $10^{-2}\,\hbox {m}$ and $10^{-1}\,\hbox {s}$.

Near-surface wind speed measurements with a short-path ultrasonic anemometer served as a reference measurement. It was shown that the turbulent sensible heat fluxes calculated from the measured data are in agreement with profiles of horizontal and vertical wind and temperature obtained from the screen measurements. Two different characteristic situations with positive and negative sensible heat fluxes and the corresponding infrared frame with vertical profiles of temperature and wind field were juxtaposed. This comparison elucidates the highly variable behaviour of the near-surface atmospheric layer and demonstrates the capability of the presented method to measure the spatio-temporal dynamics. Furthermore, a histogram comparison of horizontal and vertical velocity components over the $10\,\hbox {min}$ sequence proves the validity of the WEIRD estimations.

The results above display the power of the screen set-up in combination with WEIRD for investigation of the near-surface atmospheric layer dynamics. Such a set-up may also be useful for addressing other research questions, with the requirement of heterogeneous air temperatures at the metre-scale. Other possible applications could include the characterization of different kind of slope flows such as thermal updrafts, drainage, or katabatic flows (Grudzielanek and Cermak 2015). Especially measuring properties of low-level jets within deep katabatic flows on glaciers might be an interesting application (Sauter and Galos 2016; Mott et al. 2020). Furthermore, debris-covered glaciers or perennial ice fields offer a strong surface heterogeneity. The investigation of near-surface atmospheric flow and surface heat exchange processes might, thus, benefit from high spatio-temporal resolution screen measurements (Brock et al. 2010; Mott et al. 2019). Spatially scarce point-based eddy-covariance sensors are not capable of resolving the full near-surface vertical structure. The screen method can fill this gap with spatially continuous measurements.

In ongoing and future research, we will apply the presented methods on multiple recorded sequences in various conditions. We hope to get a broader and deeper insight into the metre- and sub-metre-scale processes present above patchy snow covers, and their consequences for snow melt in late spring. This will be studied with the aim of developing better parametrizations of these processes for use in coarse-scale snow melt models.

## Supplementary Information

Below is the link to the electronic supplementary material.

Supplementary file 1 (png 48 KB)

Supplementary file 2 (mp4 26570 KB)

Supplementary file 3 (pdf 138 KB)

## Data Availability

Data and documented source code is available at 10.16904/envidat.299.
