# Supplementary figures and images for: A Novel Method to Quantify Near-Surface Boundary-Layer Dynamics at Ultra-High Spatio-Temporal Resolution
Source: Boundary Layer Meteorol. 2022 Nov 19;186(2):177–97. doi: 10.1007/s10546-022-00752-3 (PMC9902431; doi:10.1007/s10546-022-00752-3)

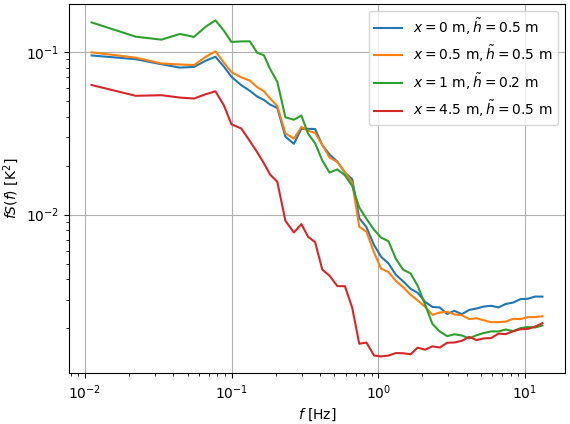

Supplement: Supplementary file 1 — (png 48 KB) [file 10546_2022_752_MOESM1_ESM.png]
