# Supplementary material for: A Novel Method to Quantify Near-Surface Boundary-Layer Dynamics at Ultra-High Spatio-Temporal Resolution
Source: Boundary Layer Meteorol. 2022 Nov 19;186(2):177–97. doi: 10.1007/s10546-022-00752-3 (PMC9902431; doi:10.1007/s10546-022-00752-3)
Supplement: Supplementary file 3 — (pdf 138 KB) [file 10546_2022_752_MOESM3_ESM.pdf]

Supplementary Material for A Novel Method to Quantify Near-Surface Boundary-Layer Dynamics at Ultra-High Spatio-Temporal Resolution

Michael Haugeneder* · **Michael Lehning** · **Tobias Jonas** · **Rebecca Mott**

*WSL-Institute for snow and avalanche research SLF, michael.haugeneder@slf.ch

Spectral Density

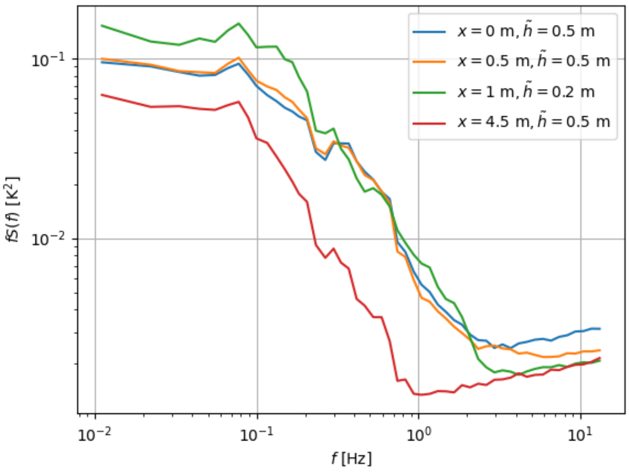

Fig. 9 Spectral density of the screen temperature time series for four different pixels. The height above snow, $\tilde{h}$, and the fetch distance x are given for the four pixels

Real Time Near-Surface Atmosphere Dynamics (Video)

A 1 min excerpt from the recorded infrared sequence (28 April 2021 1200 LT) in real time is uploaded separately. The video shows the advection of warm air plumes over the snow surface and the strong spatio-temporal dynamics of the near-surface atmospheric layer over patchy snow cover. The field of view is the same as in Fig. 4.
